# Biological variation: a still maturing aspect of laboratory medicine

**DOI:** 10.1515/almed-2019-0032

**Published:** 2020-09-25

**Authors:** Callum G. Fraser

**Affiliations:** University of Dundee, Centre for Research into Cancer Prevention and Screening, Ninewells Hospital and Medical School, Dundee, DD1 9SY, United Kingdom of Great Britain and Northern Ireland

**Keywords:** analytical performance specifications, between-subject biological variation, biological variation, reference change values, within-subject biological variation

Until relatively recently, the topic of biological variation (BV) in laboratory medicine would have seemed mature. It was well documented that, although some analytes had cyclical rhythms that could be daily, monthly or seasonal in nature, the variation of most analytes over time could be considered random [1]. The generation of numerical data on the components of BV, namely, within-subject BV, the average variation around the homeostatic setting point (CV_I_), between-subject BV, the difference among the setting points of individuals (CV_G_), had been reviewed [2]. The application of data on CV_I_ and CV_G_ in setting examination performance specifications for imprecision, bias, total error and measurement uncertainty; in deciding the significance of changes in serial results in an individual; in assessing the probability that a change in serial results is significant; in calculating the index of individuality and in examining the value of traditional population-based reference values and partitioning according to, for example, age and sex; and other uses, had all been well accepted [1].

The application of data on the components of BV throughout laboratory medicine was greatly assisted by the generation of a series of comprehensives databases of components of BV, initiated in 1997, which integrated information found in publications on BV. A scoring system was designed to ensure the robustness of the data included. Until 2014, when data on 358 analytes were documented, this incredibly valuable resource was updated every two years and then made available on the Internet [3]. However, doubts began to arise on the accuracy of certain estimates included in the databases: these were described in detail at the 1st European Federation of Clinical Chemistry and Laboratory Medicine (EFLM) Strategic Conference on “Defining analytical performance goals – 15 years after the Stockholm Conference”. It was comprehensively documented by Carobene that, although agreeing that these databases had proven to be valuable, it was clear that there were several issues that impacted their utility [4]. It was stated that what was needed was the creation of a complete database derived from appropriately powered studies delivering the numerical components of BV along with confidence intervals. Moreover, application of a checklist would enable existing data to be reassessed and drive up the quality of future reported studies. In addition, the need to deliver contemporary data could be facilitated by creation of a bank of appropriately collected specimens which would enable new and valid data sets on BV to be generated.

Through a variety of initiatives, the EFLM and other groups, particularly the Spanish Society of Laboratory Medicine, have, in the last five years, delivered a significant quantum of these desirable prerequisites to improved generation and application of data on BV [5]. These well-conducted works include the large European Biological Variation Study (EuBIVAS), which has already delivered high-quality data on BV for a wide range of measurands, using samples collected under optimal conditions, minimising pre-examination sources of variation, as well as using modern examination methodology and correct statistical analyses. Very importantly, because it would be impossible for all users of BV data to generate their own estimates and reliance on databases is recommended, critical appraisal and meta-analysis of published BV studies are now possible through application of the Biological Variation Data Critical Appraisal Checklist (BIVAC) [6]. Now, global BV estimates derived from meta-analysis of the BIVAC appraised publications are accessible in a Biological Variation Database at the EFLM website created by the EFLM Working Group on Biological Variation and the Task Group for the Biological Variation Database [7]. Currently, 488 publications are referenced, 1,782 records of data on BV are included and 143 measurands with data are listed.

In this issue of Advances in Laboratory Medicine, a well-designed and executed study examines data on BV for glucose and HbA_1c_ [8]. The critical appraisal checklist (BIVAC) [6] was applied to 40 articles, 23 concerning the BV of glucose and 17 on the BV of HbA_1c_. The BIVAC has 14 items, which classify the content of BV articles into four categories, A, B, C or D, in descending order of quality, as well documented in this study. Overall BV estimates (CV_I_ and CV_G_) were calculated by means of a meta-analysis that gave priority to articles with the highest quality scores.

Very interestingly, the new BV estimates for glucose and HbA_1c_ were very similar to those detailed in the previously created database [3]: in consequence, a germane question is whether this applies to other measurands because the new EFLM database documents only 143 measurands while the 2014 database concerns 358; perhaps this implies that users should continue to apply the data in the 2014 database with confidence at least until newer data, generated through EFLM meta-analysis, become available. Thus, potential users of BV data should first interrogate the EFLM database [7] and, if data on the measurand of interest are lacking, the data in the 2014 database [3] should be used if documented there.

However, as shown previously for other measurands [4], few articles examined in the study attained the highest quality classification, almost none contained data on the clinical stability of the subjects involved, very few validated a normal distribution of data, around 75% did not evaluate the homogeneity of variances and less than half used duplicate examination results to calculate the imprecision (CV_A_). Assessment of most of the data on BV generated to date on measurands examined in the EuBIVAS study clearly shows that the new estimates of CV_I_ and CV_G_ are generally rather smaller than those in the 2014 database, presumably because of the careful selection of apparently healthy subjects, minimisation of pre-examination variation, use of modern examination methodology with higher specificity and application of correct statistical techniques with exclusion of outliers. As stated by Ricós et al. [8], these facts do imply the need for further BV studies on measurands related to the diagnosis and monitoring of diabetes mellitus to refine the estimates of CV_I_ and CV_G_, using modern methods and following the many guidelines, recommendations and practices promulgated under the auspices of EFLM [5].

In another contribution to this issue, Díaz-Garzón et al. [9] concisely describe the models available to estimate the components of BV, document their pros and cons and suggest approaches to selecting the appropriate method for each specific application of data on BV. The models discussed encompass direct methods to estimate the components of BV using the very widely applied one developed by Fraser and Harris [2], the recently revised one by the EFLM [5], mixed-effects models and the more recent, and yet not widely applied, Bayesian model.

Because undertaking studies on BV using all these approaches is difficult and very resource consuming, particularly if done correctly using the EFLM approaches [5], there is real interest in indirect methods, based on retrospective studies using extensive results generated on samples from patients and often available in laboratory informatics systems, termed the “big data” approach. Despite the disadvantages, well listed by Díaz-Garzón et al. [9], the advantages documented include that variables such as age, gender and study duration are easily assessed, the large number of subjects compiled increases statistical power and the approach is inexpensive and does not require adjuncts such as an experimental study or ethical approval. This approach is examined in a recent study in which pairs of sequential results from adult patients were extracted from a routine pathology database for some commonly examined chemical and haematological tests [10]. Very interestingly, this approach produced estimates of CV_I_ that, for most of the 26 measurands studied, showed good agreement with published data. The estimates demonstrated minimal effect of sex, age or time between samples. Thus, extending the proposal made previously, potential users of data on BV should first interrogate the EFLM database [7] and, if data on the measurand of interest are lacking, the data in the 2014 database [3] should be used if documented there and, if data are still lacking, CV_I_ could be generated from careful documentation of the results of pairs of results in a laboratory database and the strategies proposed by Jones [10] followed.

BV is clearly still an evolving aspect of laboratory medicine, not yet totally mature. The two contributions in this issue of the journal [8, 9] do assist in progressing the generation and application of data on BV towards a more fully developed subject and support the adoption of a hierarchical approach to selection of data on the components of BV.
